# How to Measure a Two-Sided Market

**DOI:** 10.3390/e23080962

**Published:** 2021-07-27

**Authors:** Wen Zheng, Xiaoming Yao

**Affiliations:** School of Management Science, Northeastern University at Qinhuangdao, Qinhuangdao 066004, China; yaoxiaoming@neuq.edu.cn

**Keywords:** market structure entropy (MSE), O2O platform transaction, complex network, market quality entropy, market time-effect entropy, market capacity entropy

## Abstract

Applying the theories of complex network and entropy measurement to the market, the two-sided market structure is analyzed in constructing the O2O platform transaction on the entropy measurement of the nodes and links. Market structure entropy (MSE) is initially introduced to measure the consistency degree of the individuals and the groups in the O2O market, according to the interaction in the profits, the time/space, and the information relationship. Considering that the market structure entropies are changing upward or downward, MSE is used to judge the consistency degree between the individuals and the groups. Respectively, considering the scale, the cost and the value dimensions, MSE is expanded to explain the market quality entropy, the market time-effect entropy, and the market capacity entropy.MSE provides a methodology in studying the O2O platform transaction and gives the quantitative index in the evaluation of the O2O market state.

## 1. Introduction

In considering about a two-sided market, we can analyze the system in a perspective emergence in bottom-up from a micro- to macro-way. O2O platform is a typical two-sided market. Adaptive agents in O2O platform emerge in the surroundings and form the market structure. Furthermore, they co-impact and co-influence with the surroundings [1,2]. The agents are not passive receivers of the market systematic environment. They act on the market structure with the different attributes and strategies [3]. Based on the consistency degree of the individual and market group objectives, the market structure entropies are introduced as a measurement tool for analyzing the O2O platform trading systems. This paper analyzes the number of nodes in the complex network structure of platform transaction and the market structure of the nodes-lines to measure the market quality entropy of the consistency between the platform individual and the seller group, the market time-effect entropy of the consistency between the seller individual and the consumer group, and the market capacity entropy of the consistency of the information between the platform individual and the consumer group. They are used to deliver the platform transaction and the market structure state caused by the O2O platform transaction.

In O2O platform trading, a complex network that consists of the participants, their attribution nodes, and the transaction connections is expanded a $G=\{N\sim E\left(\lambda_{1}\right),(L_{x}\sim B(1,p),L_{x}\sim B(n,p^{\prime }),L_{x}\sim P(\lambda_{2})),L_{Cc}\sim N(\mu ,\sigma^{2})\}$. Among them, N indicates the number of nodes, and L indicates the link connections. Platform trading is composed of the nodes and link connections. About nodes, agents are its main body, such as consumers, sellers, and platforms. Concerning link connections, they are composed of consumer-seller (the transaction link), consumer-platform (the information link), and seller-platform (the income distribution link). The definition of market structure entropy $\mathrm{MSE}\;(\mathrm{Market}\;\mathrm{Structure}\;\mathrm{Entropy})=\{MQE\left(N\right),MTE\left(L_{x}\right),MCE(N,L_{Cc})\}$ is used to restore the market structure shaped by the links of market participants (agents) and to show the market condition of O2O platform transaction.

Market structure entropy in showing individual interaction is the base on which to judge the market condition of O2O platform trading. That is, if the order between individual and groups is consistent, the entropy decrease will appear, which forms the mutual benefit mechanism of individual utility; if the order between individual and group is inconsistent, then entropy increase will appear. Entropy increase leads to the co-harm mechanism in individual effect.

## 2. Literature Review

The entropy model [4,5,6,7], constructed by entropy measurement principles, is widely used to evaluate the system state of the market. Multi-scale sample entropy (MSEBSS) is based on similarity measure stock market stability [8]. Based on the node degree distribution, relative importance, and point-side difference of the park economic network, the entropy of Cai’s structure and Wu’s structure entropy and the entropy increases with the increase of market network scale, and the stability of the park system decreases [9]. Considering the interests of the participants in the system, it is proposed that the conflicts of interest between the members produce the system entropy increase and the poor stability of the organizational structure of the enterprises [10]. Entropy value of China’s carbon market increases with the degree of unreasonable market structure distribution [11]. In the above study, the entropy-increase is associated with the difference of system state stability.

In the research on entropy decrease in Indian stock markets, systemic entropy down from household income uniformity in European countries indicates the stable characteristics of the income economy system [12,13]. The law of entropy decrease is used to analyze the stability of the four major foreign exchange cluster centers in Europe [14]. The entropy random DEA model is used to analyze the entropy decrease of power resource allocation and power resource allocation homogenization in the power market [15]. The transferred entropy, based on the entropy research of Shannon, states that the smaller the entropy, the stronger the connectivity to the global stock market network [16]. Based on Shannon entropy, judging from the timeliness and accuracy of information flow, the evaluation model of the order degree of China Electric Power Regulatory Organization (ERI) is constructed [17]. Considering that market efficiency will develop over time [18], the time series of financial markets are turned into networks to research the effect of network entropy decrease on market efficiency [19]. These studies show that entropy decrease is associated with market benefit allocation, information resource allocation, and market efficiency consistency.

Some studies have introduced the network node degree distribution and nodes-links features to define structural entropy [20], considering the system entropy evaluation model [21] with time limitation and quality of information flow and the structure state of network organization entropy measure system involving multi-agent factors [22]. From the changes of relationship between market participants, the maximum entropy of the Markov chain studies the relationship between transferred entropy and macro-control of market economy system [23]. Statistical entropy based on market share show the power-law distribution state of competitive market structure [24].

## 3. Distribution Function

### 3.1. Exponential Distribution Function in Nodes Degree

In the process of one-to-one matching transaction, the buyer and seller produce the variable K to link the main node and other nodes. The node degree of the platform connecting two-sided market users is the number N of user nodes it connects. The platform node has the trading mechanism [25] of “connecting two-sided market user nodes-matching transaction in Nodes-Links-producing trading volume-platform node degree increasing-platform generating economic benefits, so the value of cumulative transaction volume on the platform increases exponentially with the increase of user scale index. This means that the platform in the trading center can take advantage of the network externality, can occupy more of the number of links in the network, and become the hub node in the network. The seller and the consumer in the platform lock-in conditional transaction link become most of the sub nodes in the network. The node degree distribution of platform network satisfies the power characteristics of BA scale-free network [26].

Due to the influence of network externality, the value $V_{P}$ of platform node is a positive proportional function about the square of user scale N [27], that is, $V_{P}=\alpha \cdot N^{2}$. α means the value of network to each individual. Considering the influence of positive and negative externality, the value range of α is (-∞,+∞). Growth in the number of platform user nodes N (platform node degree) follows the exponential distribution of parameters $\lambda_{1}$, that is, $N\sim E\left(\lambda_{1}\right)$, and $\lambda_{1}$ means the number of user nodes are N within each unit time, and its distribution function is

(1) $$F\left(N;\;\lambda_{1}\right)=1-e^{-\lambda_{1}N},\lambda_{1}>0$$

### 3.2. Distribution Function in Nodes-Links

#### 3.2.1. Boolean Distribution

Suppose: In O2O platform transactions, platform users get transactions through the platform. Platform nodes are distributed from 0–1, and the nodes-links of two-sided market users’ nodes (consumer-seller) are in Boolean distribution $L_{X}\sim B\left(l,p\right)$, and its distribution function is

(2) $$P\left(L_{X}=l\right)=p^{l}{\left(1-p\right)}^{1-l},l=0,1$$

Among them, the random variable $L_{X}=l$ means whether the two-sided market users reach the transaction relationship with the nodes-lines probability p. The value range of p is 0≤p≤1.

(3) $$\left\{ \begin{array}{l} l=1,\;\mathrm{users}\;\mathrm{transaction}-\mathrm{links}\;\mathrm{succeed},\;p\\ l=0,\;\mathrm{users}\;\mathrm{transaction}-\mathrm{links}\;\mathrm{failed},\;1-p \end{array}\right.$$

#### 3.2.2. Random Distribution

Suppose: In online payment-offline service of O2O platform transactions, consumers and sellers with a certain random probability p connect to form a random network. Consumers transact through the platform with online booking, payment, with electronic vouchers waiting to accept the seller’s offline services. A dynamic average path of the random, the participant books and pays with electronic vouchers, waiting to accept the seller’s offline services through the online platform. A dynamic average path of the random, considering the information transmission of O2O platform transactions, which is affected by market supply and demand, platform information, and seller cost factors, the average path length d of success of two-sided market users (both buyers and sellers) is uncertain.

The average path length d in the n node-links $L_{X}$ within per unit time, the probability of connection m times is $P^{\prime }=\left(L_{X}=m\right)$, and $L_{X}$ is taken from the natural number. For any integers m, (0≤m≤n), there is: the connection times $L_{X}$ of average path length d obey binomial distribution, that is, $L_{X}\sim B\left(n,p^{\prime }\right)$, $p^{\prime }=p\cdot \frac{d}{\sum_{i=1}^{n}d}$, and $\left(0\leq p^{\prime }\leq 1\right)$; its probability density function is as following:

(4) $$P^{\prime }\left(L_{x}=m\right)=\left(\begin{array}{l} n\\ m \end{array}\right){\left(p^{\prime }\right)}^{m}{\left(1-p^{\prime }\right)}^{n-m}$$

#### 3.2.3. Poisson Distribution

Suppose: Platform participants’ scale N→+∞, and participants’ nodes-links $L_{X}$ approximately obey the Poisson distribution $L_{X}\sim P\left(\lambda_{2}\right)$ of parameter $\lambda_{2}$. $\lambda_{2}$ means the average connection times of path length d of n nodes-links within per unit time. Its probability density function is as following:

(5) $$P\left(L_{x}=m\right)=\lambda^{m}e^{-\lambda_{2}}/m!,\;\lambda_{2}\in {\left(0,+\infty \right)}^{\;}$$

The expansion of network scale, the failure of transaction information transmission, and the uncertainty of the average path length d in the participants’ trading connection leads to the unstable characteristics of market transaction efficiency.

### 3.3. Normal Distribution Function in Nodes-Links

Platforms and sellers take advantage of the consumer preference to achieve the rapid aggregation of similar needs, which gathers the consumers with similar attributes at one end of a two-sided market in which users judge the quality of goods, prices, and other trading information of many sellers and select the best of the nearby sellers and connect them. Under the influence of cross-network effect, the two-sided market users with increasing scale finally gather in the platform node. Platform network scale growth and the preferred connection of consumers form the scale-free network structure of O2O platform trading.

As a transaction intermediary, the platform provides consumers with different types of seller information and charges consumers transaction fees. Consumers with different preferences pay different transaction costs $L_{Cc}$ to the platform. $L_{Cc}$ obeys the normal distribution, with μ as mean of transaction cost and $\sigma^{2}$ as variance of transaction cost, that is, $L_{Cc}\sim \left(\mu ,\sigma^{2}\right)$. Its probability density function is as following:

(6) $$f\left(L_{C_{c}}\right)=\frac{1}{\sqrt{2\pi }\delta }e^{\frac{{\left(L_{C_{c}}-\mu \right)}^{2}}{2\delta^{2}}},\;L_{C_{c}}\in (0,L_{C_{c}^{h}}{]}^{\;}$$

Among them, $L_{C_{c}^{h}}$ is the highest acceptable transaction cost for consumers.

## 4. Entropy in Two-Sided Market

### 4.1. Market Quality Entropy 

Definition: the time t (t≠0) of the initial state of the non-system, the number of O2O platform users, N = the number of consumers, and $N_{C}$+ the number of sellers $N_{S}$.

Hypothesis: each seller sells homogeneous goods that meet different market needs. A seller $i\left(i\in N_{S}\right)$ sells goods at a price of $p_{Si}$, and $p_{Si}=-a+bq_{Si}$ [28]. Among them, a(a>0) is the upper limit of commodity price acceptable to the consumer group, b(b<0) is the price elasticity of seller supply, and $q_{Si}$ the supply of seller’s goods.

The commodity cost function of seller i is as following:

(7) $${C_{Si}}^{\prime }=cq_{Si},c\in {\left(0,a\right)}^{\;}$$

Among them, c is the cost parameter for the seller to consider the market supply and the demand.

Assuming that the demand of consumers in the market is equal to the quantity supplied by the seller, the volume function of the cumulative number of transactions reached by the seller is as following:

(8) $$TN_{Si}=\beta_{Si}-\frac{c}{N_{S}}+\gamma {\left(p_{Si}-c\right)}^{\;}$$

Among them, β(β>0) is the ideal strategic supply quantity of the seller under the condition of considering consumer price and quality demand; γ(γ≥0) is the influence factor of platform information quality on seller’s supply quantity.

Considering the nodes-links relationship of income distribution between the seller i and the platform, the seller first pays a certain transaction fee ${C_{Si}}^{\prime \prime }$ to the platform. After starting the trading activity, the platform collects the fee ν from each transaction benefit of the seller in proportion $\omega_{p}$, then at the time t (t≠0), the total draw cost of the seller’s transaction volume is ${C_{Si}}^{\prime \prime \prime }=\nu TN_{Si}$.

The cost function of seller i is as following:

(9) $$\begin{array}{ll} C_{Si} & ={C_{Si}}^{\prime }+{C_{Si}}^{\prime \prime }+{C_{Si}}^{\prime \prime \prime }\\ & =\;cTN_{Si}+{C_{Si}}^{\prime \prime }+\nu TN_{Si} \end{array}$$

The return function of transaction volume of the seller i is as following:

(10) $$V_{Si}=p_{si}TN_{si}-C_{Si}$$

The average return on sales volume reached by the seller group is as following:

(11) $${\bar{V}}_{S}=\left(\sum_{i=1}^{N_{s}}V_{Si}\right)/N_{S}$$

The platform is linked to the seller’s revenue distribution and gets $V_{p}$ and $V_{p}=\alpha \cdot N^{2}$, and α represents the value of positive and negative network externality to individual traders, α∈(−∞,+∞).

(12) $$MQE\left(i\right)=-P_{1}\left(i\right)\log P_{1}{\left(i\right)}^{\;}$$

MQE(i) describes the inconsistency between the interest objective of seller i and the platform, and $P_{1}\left(i\right)$ is a probability function describing the offset degree between the seller’s income and the group’s average income under the influence of platform order.

(13) $$P_{1}\left(i\right)=\frac{V_{s_{i}}-{\bar{V}}_{s}}{V_{p}}$$

Among them, $V_{s_{i}}-{\bar{V}}_{s}$ describe the offset of seller i to the average income of groups and the disorder that may be caused by platform to the seller in pursuit of the profit goal. The income value of the platform $V_{p}$ increases with the increase of the number of user nodes.

Satisfying the system iteration condition of O2O platform transaction, the MQE is as follows:

(14) $$MQE={\sum_{i=1}^{Ns}MQE\left(i\right)}^{\;}$$

O2O trading nodes of the platform are exponentially distributed, and the market quality entropy is introduced to analyze the degree of inconsistency between the agent of the O2O platform and the interests of the seller group.

(15) $$MQE\left(N\right)=-{\sum_{i=1}^{N_{s}}\frac{V_{s_{i}}-{\bar{V}}_{s}}{V_{p}}\log\frac{V_{s_{i}}-{\bar{V}}_{s}}{V_{p}}}^{\;}$$

According to Formula (15), when the deviation of the seller i’s income from the average income of the group is larger, the greater the entropy value of the market quality, and the power law characteristic of the market force imbalance O2O the platform participants is systematically expressed.

Market maximum quality entropy of O2O platform trading maintains that the interaction between the platform and the seller group has an impact on the market system structure.

(16) $$MMQE=\log V_{p}$$

With the expansion of the user scale, the value of the platform network increases exponentially. It shows that the complexity of market network structure increases, and the potential risk of stability exists in a two-sided market system.

### 4.2. Market Time-Effect Entropy

Definition: The number of consumers at the time t (t≠0) of non-system initial state is $N_{c}$, the number of sellers is $N_{s}$. Consumer i with seller j matches transactions in random nodes-links probability, $i\in N_{c},j\in N_{s}$, p(0≤p≤1).

#### 4.2.1. Boolean Distribution

Definition: two-sided market users decide at time t (t≠0) whether to reach transaction nodes-links.

Meeting the user’s arbitrary trading nodes-links in condition $L_{x}\sim B\left(l,p\right)$, the market time-effect entropy is as following:

(17) $$MTE\left(L_{x}\right)={\left\{ \begin{array}{l} 0,l=0\\ -\left(p\log p\right),l=1 \end{array}\right.}^{\;}$$

l = 0 means that two-sided market users’ nodes-links transactions failed; the entropy of users in no interaction is 0. l = 1 means that two-sided users successfully link in random probability P. Entropy (-plogp) generated by interaction of agents expresses the disorder of user interaction to platform structure.

#### 4.2.2. Random Distribution

Definition: Consumer i and seller j at the time t (t≠0) link in random probability P and produce the average path length $d_{ij}\in \left[0,d_{ij}^{h}\right]$. $d_{ij}^{h}$ is the maximum average path length for two-sided market users.

(18) $$MTE\left(ij\right)=-P_{2}\left(ij\right)\log P_{2}{\left(ij\right)}^{\;}$$

MTE(ij) describes the time-space uncertainty of consumer i and seller j when they exchange the nodes-links information. $P_{2}\left(ij\right)$ is the average path $d_{ij}$ of users i and j and its probability of total average path of the finite nodes-lines between $N_{C}$ consumers and $N_{s}$ sellers.

(19) $$P_{2}\left(ij\right)=p\cdot \frac{d_{ij}}{\sum_{i=1}^{Nc}\sum_{j=1}^{Ns}d_{ij}}$$

Among them, $d_{ij}$ expresses the transaction goods and time-space distance of passing service information. Sum of average paths $\sum_{i=1}^{Nc}\sum_{j=1}^{Ns}d_{ij}$ for two-sided users is increasing with the increase of nodes and links.

Hypothesis: The average path length $d_{ij}$ (consumer waiting time $T_{C}$ or spatial distance M) of the transaction links between consumer i and seller j at the time t (t≠0) is proportional to market demand D and inversely proportional to the seller service cost $C_{Sf}$ and inversely proportional to the O2O platform information service level $\eta_{P}$.

Then the waiting time (spatial distance) function of the consumer is

(20) $$T_{c}=M_{c}=b_{1}D-b_{2}C_{Sf}-b_{3}\eta_{P}=f{\left(D,C_{Sf},\eta_{p}\right)}^{\;}$$

Among them, $b_{1}>0$ is the sensitivity factor of waiting time (space distance) to demand; $b_{2}>0$ is the sensitive factor of waiting time (space distance) to the seller’s service cost; $b_{3}>0$ is the sensitive factor of waiting time (space distance) to platform information service. The demand positively affects the seller’s service cost, and the increasing of demand and the seller’s service cost make the platform improve the information service level and make the consumer demand sensitive to commodity price [29].

There is: $\eta_{P}=f\left(D,C_{Sf}\right)$, $C_{Sf}=f\left(D\right)$, $D=f\left(p_{si}\right)$.

Hypothesis: About online and offline O2O platform trading in unit time from t→t+1 (t≠0), the individual consumer produces a unit of demand and group demand $D=D_{1}\cup D_{2}$. $D_{1}$ is the online booking demand for consumers; $D_{2}$ is the offline demand for consumers. They obey the normal distribution of the interval [A,B], 0≤A<B.

Then, the waiting time (space distance) of the individual consumer is as following:

(21) $$\begin{array}{ll} d_{ij} & =∭f\left(D,C_{Sf},\eta_{P}\right)dDdC_{Sf}d\eta_{P}\\ & =\int_{0}^{1}f\left(D_{1}\right)dD_{1}=\int_{0}^{1}f\left(D_{2}\right)dD_{2} \end{array}$$

The total waiting time (spatial distance) of the consumer group is as following:

(22) $$\begin{array}{ll} \sum_{i=1}^{Nc}\sum_{j=1}^{Ns}d_{ij} & =∭f\left(D,C_{Sf},\eta_{P}\right)dDdC_{Sf}d\eta_{P}\\ & =\int_{A}^{B}f\left(D_{1}\right)dD_{1}+\int_{A}^{B}f\left(D_{2}\right)dD_{2} \end{array}$$

Satisfying the system iteration condition of O2O platform transaction, the market time-effect entropy is as following:

(23) $$MTE={\sum_{i=1}^{N_{c}}\sum_{j=1}^{Ns}MTE\left(ij\right)}^{\;}$$

In a nodes-links in randomly distributed O2O platform deals, the market time-effect entropy MTE is introduced to analyze the space-time consistency of the user’s information nodes-links.

(24) $$MTE\left(L_{x}\right)=-{\sum_{i=1}^{N_{c}}\sum_{j=1}^{N_{s}}\left(p\cdot \frac{d_{ij}}{\sum_{i=1}^{N_{c}}\sum_{j=1}^{N_{s}}d_{ij}}\right)\log\left(p\cdot \frac{d_{ij}}{\sum_{i=1}^{N_{c}}\sum_{j=1}^{N_{s}}d_{ij}}\right)}^{\;}$$

The maximum market time-effect entropy of the O2O platform trading is:

(25) $$MMTE=\log\int_{A}^{B}f\left(D\right)dD^{\;}$$

#### 4.2.3. Poisson Distribution

With t→∞(t≠0), $N_{C}\to \infty ,N_{S}\to \infty$, in unit time, two-sided market participants produce infinite n(n→∞) transaction nodes-links, and in the n nodes-links, $nP^{\prime }$ average path lengths $d_{ij}$ are connected($P^{\prime }$ is the linking probability of average path length $d_{ij}$ in infinite path).

Hypothesis: Within each unit of time t→t+1 (t≠0), market demand does not exceed the threshold κ(κ>0). Then, the sum of the average path length of the infinite nodes-links of the two-sided market users is as following:

(26) $$\begin{array}{l} \underset{\begin{array}{l} N_{c}\to \infty \\ Ns\to \infty \end{array}}{\lim}\sum_{i}^{N_{c}}\sum_{j}^{Ns}d_{ij}\\ =∭f\left(D,C_{Sf},\eta_{P}\right)dDdC_{Sf}d\eta_{P}\\ =\int_{A}^{\kappa }f\left(D\right)dD=\int_{A}^{\kappa }f\left(D_{1}\right)dD_{1}+\int_{A}^{\kappa }f\left(D_{2}\right)dD_{2} \end{array}$$

Based on the O2O platform trading nodes-links obeying Poisson distribution, the market efficiency entropy MTE is introduced to analyze the uncertainty of the circulation of space-time transaction information among users. Its function is as following:

(27) $$MTE\left(L_{x}\right)=-{\sum_{i=1}^{N_{c}}\sum_{j=1}^{N_{s}}\left(\frac{nP^{\prime }\cdot p\cdot d_{ij}}{\int_{A}^{\kappa }f\left(D\right)dD}\right)\log\left(\frac{p\cdot d_{ij}}{\int_{A}^{\kappa }f\left(D\right)dD}\right)}^{\;}$$

From the Formula (27), it can be seen that the longer the arbitrary average path $d_{ij}$, the greater the market time-effect entropy and the more unstable the characteristics of market transaction efficiency are.

The maximum market time-effect entropy of the O2O platform transaction is as following:

(28) $$MMTE=\log\int_{A}^{\kappa }f\left(D\right)dD^{\;}$$

For the Formula (28), the larger the value of time-effect entropy in a two-sided market is, the longer the waiting time (space distance) of consumers $\int_{A}^{\kappa }f\left(D\right)dD$, and the higher the complexity of information transmission in platform transactionare.

### 4.3. Market Capacity Entropy

Definition: At the time t (t≠0) of the non-systematically initial state, consumer i pays the transaction cost $C_{c_{i}}$ to platform for its transaction nodes-links. The highest transaction cost they can accept is $C_{c_{i}}^{h}$. The average cost of $N_{c}$ consumers is ${\bar{C}}_{c}$. Under the condition of consumers’ transaction cost at $L_{C_{c}}\text{\textasciitilde{}}N\left(\mu ,\sigma^{2}\right)$, ${\bar{C}}_{c}=\mu$ and $C_{c_{i}}\in \left(0,C_{c_{i}}^{h}\right]$.

(29) $$MCE\left(i\right)=-P_{3}\left(i\right)\log P_{3}{\left(i\right)}^{\;}$$

MCE(i) describes the inconsistency between the behavior order of the platform and the order of consumers to obtain transaction information and pay transaction cost by providing transaction information and collecting transaction fees. $P_{3}\left(ij\right)$ describes the probability function of the deviation between individual consumer transaction cost and groups average transaction cost.

(30) $$P_{3}\left(i\right)=\frac{C_{c_{i}}-{\bar{C}}_{c}}{\sum_{i=1}^{N_{c}}C_{c_{i}}}$$

Among them, $C_{c_{i}}-{\bar{C}}_{c}$ describes the degree of deviation between consumer transaction cost and groups average cost under the influence of platform subject. The sum of transaction costs of consumer groups $\sum_{i=1}^{N_{c}}C_{c_{i}}$ is also the sum of transaction cost charged by the platform $F_{p}$, which increases with the increase of consumer trading nodes-links and trading intensity.

Satisfying the system iteration conditions of O2O platform trading, the MCE is as following:

(31) $$MCE={\sum_{i=1}^{N_{c}}MCE\left(i\right)}^{\;}$$

According to the normal distribution of O2O platform trading nodes and links, the market capacity entropy is introduced to analyze the adaptability of consumers to the market trading environment. The more sensitive consumers are to environmental changes, the higher their ability is and the smaller the entropy of ability to interact with the platform.

(32) $$MCE\left(N_{c},L_{C_{c}}\right)=-{\sum_{i=1}^{N_{c}}\frac{C_{c_{i}}-{\bar{C}}_{c}}{F_{p}}\log\frac{C_{c_{i}}-{\bar{C}}_{c}}{F_{p}}}^{\;}$$

According to (32), the conditions for platform and consumers have information nodes-links, and the platform collects the transaction fees. The greater the deviation between the transaction cost of the consumer i and the average cost of the groups, the greater the value of the market capacity entropy, which indicates that the market interest of the platform transaction is not balanced.

The maximum market capacity entropy in O2O platform is as following:

(33) $$MMCE=\log\sum_{i=1}^{N_{c}}C_{C_{i}}=\log F_{p}$$

If the transaction cost of consumers is more, the greater the complexity of the O2O platform to provide the market trading environment, causing potential risk of the stability of the market system.

### 4.4. Remarks in Market Structure

According to (15) and (16), (28) and (29), and (32) and (33), the inconsistency degree of individual and group interests, space/time, and information order of O2O platform transaction described by market quality entropy MQE and maximum market quality entropy MMQE, market time-effect entropy MTE and maximum market time-effect entropy MMTE, market capacity entropy MCE, and maximum market capacity entropy MMCE are obtained.

To express the instability degree of the market system $\bar{MR_{i}}$ it is to replenishthe degree of stability of the market system $MR_{i}$).

(34) $$\bar{MR_{i}}=\frac{MSE_{i}}{MMSE}$$

Calculation of market quality entropy MQE/MMQE is to characterize the structural stability of O2O platform trading system $MR_{1}$.

(35) $$MR_{1}=1-MQE/MMQE,MR_{1}\in [0,1{]}^{\;}$$

Calculation of market time-effect entropy MTE/MMTE is to characterize the information stability of O2O platform trading system $MR_{2}$.

(36) $$MR_{2}=1-MTE/MMTE,MR_{2}\in [0,1{]}^{\;}$$

Calculation of market capacity entropy MCE/MMCE is to characterize the stability of O2O platform trading system $MR_{3}$.

(37) $$MR_{3}=1-MCE/MMCE,MR_{3}\in [0,1{]}^{\;}$$

In summary, market structure entropy MSE is a systematic construction from bottom to top. By measuring the degree of consistency between individual and groups order, the stable development degree $MR_{i}$ of market system is obtained.

(38) $$MR_{i}=1-\;\bar{MR_{i}}$$

According to Formula (38), the entropy decrease and entropy increase of the market structure can be quantitatively characterized as the stable or unstable state of the market system of the O2O platform.

The stability degree of market structure $MR_{1}$, information $MR_{2}$, and capacity $MR_{3}$ of the system to measure the consistency among the individual and the group in the interests, the time/space, and the information relationship, respectively, and the stability of the market system MR is as following:

(39) $$MR={\{MR_{1},MR_{2},MR_{3}\}}^{\;}$$

## 5. Application Example

Considering the Meituan App as a typical O2O platform, take the MQE as a calculation example. Select the real-time transaction data (1 March 2021 to 31 May 2021) and calculate the MQE (market quality entropy) for application analysis. According to the data of the heat map in the one-kilometer takeout service around Northeastern University Qinhuangdao in China, the statistics show that the majority of the orders are meals. Therefore, this article applies the Houyi collector to capture the monthly sales data of 20 sellers in the Meituan App for three months (1 March 2021 to 31 May 2021) in the circle of 3 km around the campus. Sales data are organized into the data group shown in Table 1. Each array is displayed in the form of sellers and their monthly sales (seller name, monthly sales).

Considering the individual transaction number of sellers and the cumulative transaction number of the platform, it can express the results of the complex interest interaction of the participants by using the transaction number of the agents to represent their benefit and measure the market quality entropy of the Meituana App in March, April, and May, respectively.

In March 2021, the average monthly transaction number of 20 sellers is 676, and the total monthly transaction number is 13,519. The MQE is as following:

$$\begin{array}{ll} MQE_{Mar} & =-\sum_{i=1}^{N_{s}}\frac{V_{Si}-{\bar{V}}_{S}}{V_{P}}\log\frac{V_{Si}-{\bar{V}}_{S}}{V_{P}}\\ & =-\sum_{i=1}^{N_{s}}\frac{TN_{Si}-\bar{T}{\bar{N}}_{S}}{TN_{P}}\log\frac{TN_{Si}-\bar{T}{\bar{N}}_{S}}{TN_{P}}\\ & =-\sum_{i=1}^{N_{s}}\frac{TN_{Si}-676}{13,519}\log\frac{TN_{Si}-676}{13,519}\;\\ & =0.464 \end{array}$$

In April 2021, the average monthly transaction number of 20 sellers is 1011, and the total monthly transaction number is 20,312. The MQE is as following:

$$\begin{array}{ll} MQE_{Apr} & =-\sum_{i=1}^{N_{s}}\frac{V_{Si}-{\bar{V}}_{S}}{V_{P}}\log\frac{V_{Si}-{\bar{V}}_{S}}{V_{P}}\\ & =-\sum_{i=1}^{N_{s}}\frac{TN_{Si}-\bar{T}{\bar{N}}_{S}}{TN_{P}}\log\frac{TN_{Si}-\bar{T}{\bar{N}}_{S}}{TN_{P}}\\ & =-\sum_{i=1}^{N_{s}}\frac{TN_{Si}-1011}{20,312}\log\frac{TN_{Si}-1011}{20,312}\;\\ & =0.512 \end{array}$$

In May 2021, the average monthly transaction number of 20 sellers is 1398, and the total monthly transaction number is 25,967. The MQE is as following:

$$\begin{array}{ll} MQE_{May} & =-\sum_{i=1}^{N_{s}}\frac{V_{Si}-{\bar{V}}_{S}}{V_{P}}\log\frac{V_{Si}-{\bar{V}}_{S}}{V_{P}}\\ & =-\sum_{i=1}^{N_{s}}\frac{TN_{Si}-\bar{T}{\bar{N}}_{S}}{TN_{P}}\log\frac{TN_{Si}-\bar{T}{\bar{N}}_{S}}{TN_{P}}\\ & =-\sum_{i=1}^{N_{s}}\frac{TN_{Si}-1398}{25,967}\log\frac{TN_{Si}-1398}{25,967}\;\\ & =0.542 \end{array}$$

The MMQE(maximum market quality entropy) is calculated as following:

Considering that the Meituan App occupies nearly 80% of the market user scale, the network externality is considered as 0.8.

In March 2021, the platform node degree is 13,519 of the total transaction number. The commission rate of the Meituan App is adjusted to 20% due to the impact of the epidemic. The s*A*ccess (platform access fee) for sellers to enter the platform is RMB 6800.00 Yuan, and 20 sellers earn an average of RMB 30 Yuan per order. The MMQE value is as following:

$$\begin{array}{ll} MMQE_{Mar} & =\log V_{P}=\log\left(externality\cdot \mathrm{platform}\_\mathrm{Degree}\cdot (sA\mathrm{ccess}+30\cdot e\mathrm{mployRate}\right)\\ & =\log\left[0.8\cdot 13,519\cdot \left(6800+30\cdot 20\%\right)\right]\\ & =7.867 \end{array}$$

In April 2021, the platform node degree is 20,312 of the total monthly transaction number, the average commission rate paid by 20 sellers is 22%, and the seller’s s*A*ccess is RMB 6800.00 Yuan. The MMQE value is as following:

$$\begin{array}{ll} MMQE_{Apr} & =\log V_{P}=\log\left(externality\cdot \mathrm{platform}\_\mathrm{Degree}\cdot (sA\mathrm{ccess}+30\cdot e\mathrm{mployRate}\right)\\ & =\log\left[0.8\cdot 20,312\cdot \left(6800+30\cdot 22\%\right)\right]\\ & =8.044 \end{array}$$

In May 2021, the platform node degree is 25,967 of the total monthly transaction number. The Meituan App implemented a new take-out commission rate policy (the commission rate is composed of a fixed technical service fee and a dynamic performance service fee affected by the order price, delivery distance, and delivery time). The average commission rate paid by 20 sellers reaches to 30%.The platform access fee (s*A*ccess) is RMB 6800.00 Yuan. The MMQE value is as following:

$$\begin{array}{ll} MMQE_{May} & =\log V_{P}=\log\left(externality\cdot \mathrm{platform}\_\mathrm{Degree}\cdot (sA\mathrm{ccess}+30\cdot e\mathrm{mployRate}\right)\\ & =\log\left[0.8\cdot 25,967\cdot \left(6800+30\cdot 30\%\right)\right]\\ & =8.151 \end{array}$$

In an empirical calculation, the MQE values of the Meituan App’s transactions in March, April, and May 2021 are shown in Table 2.

## 6. Conclusions

The complex network node degree distribution and the transaction nodes-links ofO2O platform transaction obey multiple distributions. Market quality entropy, market time-effect entropy, and market capacity entropy are proposed to measure the consistency of individuals and groups in interest, the time/space, and the information relationship. For O2O platform trading market structure analysis, it provides a new method.

## Figures and Tables

**Table 1:** The table of the sellers’ data.

March	April	May
(Mahida Rice Cake, 281)	(Mahida Rice Cake, 591)	(Mahida Rice Cake, 894)
(Liuheshun Copper Hot Pot, 317)	(Liuheshun Copper Hot Pot, 736)	(Liuheshun Copper Hot Pot, 982)
(Abilee Buffet, 684)	(Abilee Buffet, 1273)	(Abilee Buffet, 2031)
(Manyuan Spring Cake, 752)	(Manyuan Spring Cake, 1091)	(Manyuan Spring Cake, 1823)
(He Mi Tang, 867)	(He Mi Tang, 1526)	(He Mi Tang, 1743)
(Chushan Rice Ball, 305)	(Chushan Rice Ball, 747)	(Chushan Rice Ball, 869)
(Sambo Wonton, 25)	(Sambo Wonton, 112)	(Sambo Wonton, 265)
(Ribs rice, 104)	(Ribs rice, 429)	(Ribs rice, 824)
(Curry Korean Fried Chicken, 553)	(Curry Korean Fried Chicken, 1084)	(Curry Korean Fried Chicken, 2528)
(Takifukui, 694)	(Takifukui, 1305)	(Takifukui, 2473)
(Yuelai Fast Food, 1983)	(Yuelai Fast Food, 259)	(Yuelai Fast Food, 2779)
(Old Liu Xiaowancai, 261)	(Old Liu Xiaowancai, 304)	(Old Liu Xiaowancai, 440)
(Large bowl rice, 2596)	(Large bowl rice, 3281)	(Large bowl rice, 1854)
(Chopsticks Zhixiang, 487)	(Chopsticks Zhixiang, 561)	(Chopsticks Zhixiang, 833)
(Jinjia Snacks, 493)	(Jinjia Snacks, 529)	(Jinjia Snacks, 816)
(Shifu Spare Ribs, 542)	(Shifu Spare Ribs, 669)	(Shifu Spare Ribs, 911)
(Xie’s small bowl of dishes, 895)	(Xie’s small bowl of dishes, 1124)	(Xie’s small bowl of dishes, 948)
(Akushima, 974)	(Akushima, 1261)	(Akushima, 1370)
(Yunxi big bowl of rice, 337)	(Yunxi big bowl of rice, 556)	(Yunxi big bowl of rice, 491)
(Monster Fried Rice, 369)	(Monster Fried Rice, 542)	(Monster Fried Rice, 560)
Monthly sales: 13,519	Monthly sales: 20,312	Monthly sales: 25,967
Average monthly sales: 676	Average monthly sales: 1011	Average monthly sales: 1398

**Table 2:** The measurement result of MQE in the Meituan App.

	Month	March	April	May
MQE				
MQE		0.464	0.512	0.542
MMQE		7.867	8.044	8.151

## Data Availability

No new data is created in this study. Data sharing is not applicable to this article.
